# Mortality Fractions Attributable to Smoking and Smokeless and Mixed Tobacco Use Among Men and Women Across India, 1998–2021

**DOI:** 10.1093/ntr/ntaf121

**Published:** 2025-06-14

**Authors:** Rufi Shaikh, Tobias Vogt, Nandita Saikia, Mangesh S Pednekar, Prakash C Gupta, Fanny Janssen

**Affiliations:** Department of Public Health and Mortality, International Institute for Population Sciences (IIPS), Mumbai, India; Population Research Centre, Faculty of Spatial Science, University of Groningen, Groningen, The Netherlands; Population Research Centre, Faculty of Spatial Science, University of Groningen, Groningen, The Netherlands; Max Planck Institute for Demographic Research, Rostock, Germany; Prasanna School of Public Health, Manipal Academy of Higher Education, Manipal, India; Department of Public Health and Mortality, International Institute for Population Sciences (IIPS), Mumbai, India; Healis Sekhsaria Institute for Public Health, Mumbai, India; Healis Sekhsaria Institute for Public Health, Mumbai, India; Population Research Centre, Faculty of Spatial Science, University of Groningen, Groningen, The Netherlands; Netherlands Interdisciplinary Demographic Institute-KNAW and University of Groningen, Groningen, The Netherlands

## Abstract

**Introduction:**

Evidence on the mortality burden of tobacco use remains fragmented for low- and middle-income countries like India, and does not fully use Indian-specific datasets. We estimated mortality fractions attributable to different tobacco types (smoked, smokeless, and mixed tobacco use) for India by sex and state-and-union territories over time.

**Aims and Methods:**

We applied a direct prevalence approach to estimate mortality fractions attributable to tobacco types among men (35–54 years) and women (35–49 years) over time across 36 Indian states. We used national- and state-level prevalence estimates from the National Family Health Survey (1998–1999, 2005–2006, 2015–2016, and 2019–2021) and estimated Indian-specific relative risks (RRs) of all-cause mortality by tobacco type and sex by applying Cox proportional hazards models to data from the Mumbai Cohort Study.

**Results:**

RRs and sex differences therein differed by tobacco use type. Smoking exhibited the highest RR among men, while mixed tobacco use was highest among women. In 2019–2021, 45.7% and 2.5% of all deaths among Indian men and women, respectively, were related to tobacco use, driven by smoking-attributable mortality among men (28%) and smokeless tobacco-attributable mortality among women (2.1%). Tobacco-attributable mortality shares declined between 1998–1999 and 2019–2021, more strongly for women than men, with an increase in different tobacco types for men and in smoking for women until 2005–2008. State differences in tobacco-attributable mortality shares varied by sex and tobacco types, with higher shares for smoking in the Northeast region, and for smokeless tobacco in East India.

**Conclusions:**

Levels, sex and state differences, and time trends in mortality fractions attributable to tobacco use in India differed substantially by tobacco type.

**Implications:**

Our findings highlight the importance of further strengthening tobacco control initiatives by shifting to a target-oriented approach comprising different actions for each tobacco use type, aimed particularly at men and the Northeast Indian states, to enable India to achieve its Sustainable Development Goals by 2030.

## Introduction

The tobacco epidemic is one of the biggest public health threats, killing over nine million people annually.^1^ An estimated 52.2% of global deaths attributable to tobacco smoking occurred in four countries: China, India, the United States, and Russia.^2^ Although the risk of tobacco use (mostly cigarette smoking) is extensively documented for high-income countries (HICs),^3^^,^^4^ evidence on the mortality burden associated with different tobacco use types (smokeless and mixed tobacco use) for low- and middle-income countries (LMICs) like India remains fragmented.^5^^,^^6^ This knowledge is essential for monitoring and designing appropriate interventions in LMICs. While cigarette smoking is the most common form of tobacco use in HICs,^7^^,^^8^ approximately 80% of smokeless tobacco burden falls on LMICs.^9^ According to the World Health Organization (WHO), there is no safe level of exposure to tobacco, regardless of whether it is smoked, inhaled, sniffed, sucked, applied, or chewed; whether some of the harmful ingredients are reduced; or whether it is mixed with other ingredients.^7^

India is the second-largest producer and consumer of tobacco, where tobacco is consumed in many different forms (smoked and smokeless), and cigarette smoking (10.7% in 2017–2018)^10^ makes up only a small portion of overall consumption.^11^ Shaikh et al. (2022)^12^ estimated for 2015–2016, the prevalence of overall tobacco at 27% among men and 2.5% among women, smoked tobacco at 12% among men and 0.1% among women, and smokeless tobacco at 15% among men and 2.4% among women. Kaur et al.^6^ recently estimated that India accounts for 63.9% of tobacco attributable deaths in the South-East Asian region during 2019–2021, with 1.9 million deaths among males and 0.6 million deaths among females.

Most previous population-level studies for India, however, estimated smoking-attributable mortality (and not broader tobacco-attributable mortality), did not provide estimates by tobacco use type or by Indian states, or used data that were not (fully) Indian specific. The Global Burden of Disease (GBD)^2^ study estimated that in India in 2015, 13.8% and 3.1% of all deaths among men and women above age 10, respectively, were due to tobacco smoking, based on data that were not Indian-specific. Jha et al. in 2008 estimated one million deaths attributable to smoking in India in 2010 for both sexes combined.^13^ However, similar to Kaur et al., they used relative risk (RR) estimates derived from the American Cancer Prevention Study (CPS) II study. All these studies did not provide estimates by Indian state.

Estimates of mortality attributable to different tobacco use types are important because of known differences in adverse health effects by the type of tobacco consumed. Individual-level studies conducted in urban Mumbai^14^ found a two-fold higher RR of death among smokers than among non-smokers. The age-adjusted RR for all-cause mortality among men was 1.39 for cigarette smoking and 1.78 for beedi smoking (locally handmade manufactured cigarettes filled with tobacco flake and wrapped in a tendu leaf).^15^ However, risks associated with the concurrent use of smoked and smokeless tobacco (hereafter referred to as mixed-use) remain unknown. This is important because approximately 10% of smokeless tobacco users also smoke cigarettes.^16^

When monitoring tobacco-attributable mortality by tobacco type in India, it is essential to pay attention to potential sex differences, state differences, and trends over time. For example, while tobacco use declined across major Indian states, an increase in the prevalence of smoked and smokeless tobacco use has been observed in a few states of Northeast and West India.^10^^,^^11^ There are also states in Northeast India with extremely high tobacco consumption levels, comparable to those in HICs. In addition, tobacco consumption is substantially higher among men than women.^11^ While men smoke more, smokeless tobacco is the main form of tobacco among women.^10^

Our objective is to estimate, for the first time, mortality fractions attributable to total tobacco use and tobacco use type (smoked, smokeless, and mixed tobacco use) for India by sex and state, and over time. These estimates will be helpful for monitoring and designing future tobacco control and prevention interventions in India. For our analysis, we use detailed prevalence data (by sex and state) obtained from multiple rounds of the large-scale, representative, population-based Demographic Health Survey (DHS) for India, and use Indian-sex-specific RR estimates by tobacco use type.

## Data and Methods

Information on tobacco consumption by tobacco type and sex for India as a whole and for its 36 states and union territories was derived from the National Family Health Survey (NFHS), conducted in 1998–1999 (round 2), 2005–2006 (round 3), 2015–2016 (round 4), and 2019–2021 (round 5), based on self-reported tobacco use for males aged 15–54 and females aged 35–49. Based on internationally standardized questions for tobacco, we distinguished between smoked, smokeless, and mixed tobacco use. We defined individuals as smokers if they only smoked cigarettes or bidis, and did not use any other form of smokeless tobacco. Smokeless tobacco users were defined as those who consumed tobacco orally or applied tobacco on teeth or gums, and had never used smoked tobacco. Lastly, individuals who consumed both smoked and smokeless tobacco were classified as mixed tobacco users. We created mutually exclusive groups for tobacco types to ascertain the mortality fractions attributable to their use. State-specific sampling weights were applied to estimate the prevalence of each tobacco type by sex and state, and over time.

We estimated Indian-specific RRs of dying (all-cause mortality) due to different tobacco types, by sex, based on data from the Mumbai Cohort Study (MCS).^14^ The MCS, which was conducted in urban Mumbai city, is the only cohort study in India that can be used for estimating mortality due to different tobacco types (smoked, smokeless and mixed-use) among men and women. A total of 148 173 men and women over age 34 were recruited using the Mumbai election voters’ list as the selection frame. A baseline survey was conducted between 1991 and 1994 using a house-to-house approach, and the first follow-up was conducted after 5.5 years. Respondents were interviewed and classified according to their present and past tobacco use as: (1) having never used tobacco; (2) ex-smoker; (3) ex-smokeless tobacco user; (4) ever smoker; (5) ever smokeless tobacco user; and (6) ever mixed user. The endpoint (event) was death. We used a Cox proportional hazards regression model^14^^,^^17^ controlling for education (categorized as illiterate, primary, middle, secondary, and college level) as a socioeconomic variable. We estimated the RRs separately by tobacco use type (smoked, smokeless, mixed-use) and by sex. While the model chosen estimates hazards of tobacco, we will favor the term “risk” to avoid confusion further in the manuscript. (Please see the Supplementary Data and Methods file for more details.)

To estimate the share of all-cause mortality attributable to tobacco types, we used a direct prevalence approach (as opposed to the indirect approach by Peto et al.^3^^,^^4^) by applying the population-attributable fraction equation developed by Levin^17^ to the NFHS prevalence data and the all-cause mortality risks from the MCS:


(1) \begin{equation*} {\mathrm{TAMF}}_{s,c,t,y}={p}_{s,c,t,y}\left({\mathrm{RR}}_{s,t}-1\right)/\left[1+\left({p}_{s,c,t,y}\left({\mathrm{RR}}_{s,t}-1\right)\right)\right] \end{equation*}

where TAMF is tobacco-attributable mortality fractions for ages 35–54 for men and ages 35–49 for women, *p* represents prevalence of tobacco consumption (smoked, smokeless, and mixed tobacco use), *s* represents sex (men and women), *c* represents context (India or one of the 36 individual states), *t* represents type of tobacco consumed (smoked, smokeless, and mixed tobacco use), *y* represents years (1998–1999, 2005–2006, 2015–2016, and 2019–2021), and *RR* refers to the relative risk of dying by tobacco type compared to never tobacco users. We estimated TAMF separately by sex, tobacco type, and Indian state and union territory for the years 1998–1999, 2005–2006, 2015–2016, and 2019–2021. We then summed the fractions by tobacco use type to obtain mortality fractions attributable to total tobacco use, thereby accounting for the fact that the RR for tobacco use based on the MCS depends on the relative importance of smoking versus smokeless tobacco use versus mixed-use (in terms of prevalence). Since the completeness and quality of cause-specific mortality data varied across Indian states and by sex, we estimated tobacco-attributable mortality fractions for all-cause mortality and not for separate causes of death, in line with many previous studies.^18^^,^^19^

The Cox proportional hazards model was analyzed using Stata 16 software at a 5% level of significance, whereas Microsoft Excel 11 was used to estimate TAMFs.

## Results

Compared to never tobacco users, men (35–54 years) consuming tobacco in any form in India had a 67% higher risk of death, while women (35–49 years) had a 37% higher risk (Table 1). While RR for death from smoking was twice as high for men (RR: 2.48) as for women (RR: 1.44), it was identical for smokeless and mixed tobacco use.

**Table 1:** Relative risk estimates for all-cause mortality attributable to the use of different tobacco products among men (35–54 years) and women (35–49 years) in India *compared to non-tobacco users*, based on data from the Mumbai Cohort Study (MCS) over the years 1991–2005

Type of tobacco consumed	Relative risk (95% CI)	
	Men (35–54 years)	Women (35–49 years)
Total tobacco	1.67 (1.42–1.96)^*^	1.37 (1.12–1.67)^*^
Smoking	2.48 (2.05–2.99)^*^	1.44 (0.2–10.33)
Smokeless tobacco	1.35 (1.14–1.6)^*^	1.37 (1.12–1.67)^*^
Mixed use	1.64 (1.32–2.03)^*^	1.63 (0.23–11.66)

^*^ *p*-value: 5% level of significance.

**Figure 1:**
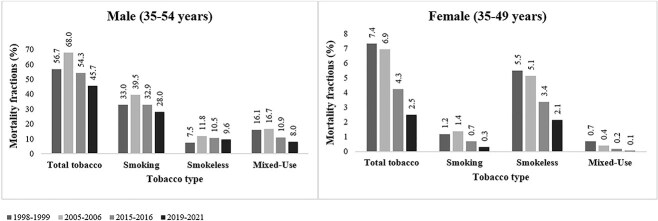
Mortality fractions (%) attributable to tobacco types* among men (35–54 years) and women (35–49 years), India, 1998–1999, 2005–2009, 2015–2016, and 2019–2021. ^*^Note: Mortality fractions attributable to total tobacco use are the combined mortality fractions due to smoking and smokeless and mixed tobacco use. For number of deaths attributable to tobacco consumption, refer to Tables S4 and S5.

In India during 2019–2021, 45.7% of all deaths among men aged 35–54 and 2.5% of all deaths among women aged 35–49 (Figure 1) were related to tobacco use. While smoking accounted for higher mortality fractions than smokeless tobacco among men, the opposite pattern was seen in women. Mortality fractions attributable to mixed tobacco use during 2019–2021 were 8% among men and 0.1% among women.

**Figure 2:**
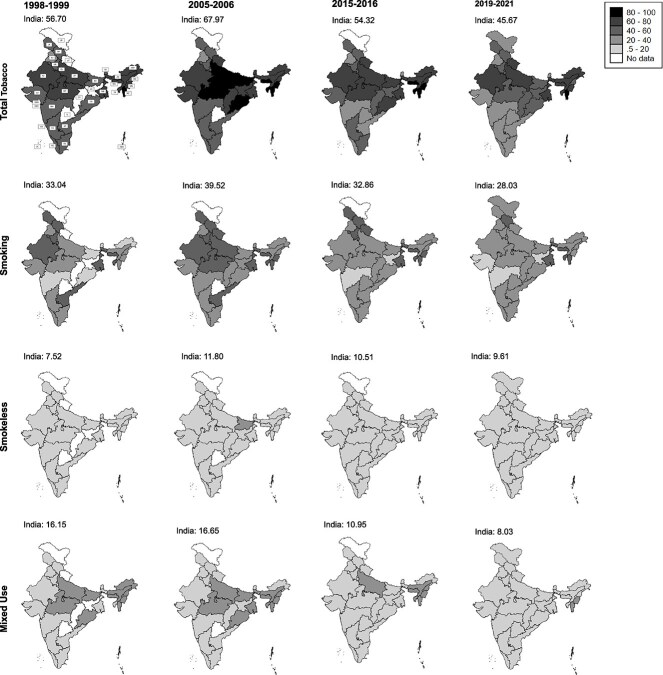
State differences in mortality fractions (%) attributable to tobacco types among men aged 35–54 years, India, 1998–2021^*^. ^*^Note: Total tobacco = sum (smoking, smokeless, and mixed use). State abbreviations: A&N = Andaman & Nicobar Islands, AP = Andhra Pradesh, ARP = Arunachal Pradesh, AS = Assam, BH = Bihar, CH = Chhattisgarh, D&N = Dadra & Nagar Haveli, D&D = Daman & Diu, DL = Delhi, GO = Goa, GJ = Gujarat, HR = Haryana, HP = Himachal Pradesh, JK = Jammu Kashmir, KA = Karnataka, KE = Kerala, LD = Ladakh, LK = Lakshadweep, MP = Madhya Pradesh, MA = Maharashtra, MN = Manipur, MG = Meghalaya, MZ = Mizoram, NG = Nagaland, OD = Odisha, PJ = Punjab, RJ = Rajasthan, SK = Sikkim, TN = Tamil Nadu, TL = Telangana, UP = Uttar Pradesh, UT = Uttarakhand, and WB = West Bengal. Prevalence of tobacco was unavailable for Uttarakhand (1998–1999), Chandigarh (1998–1999 and 2005–2006), Ladakh (1998–1999, 2005–2006, and 2015–2016), Jharkhand (1998–1999), Chhattisgarh (1998–1999), Dadra & Nagar Haveli (1998–1999 and 2005–2006), Daman & Diu (1998–1999 and 2005–2006), Lakshadweep (1998–1999 and 2005–2006), Puducherry (1998–1999 and 2005–2006), Andaman & Nicobar Islands (1998–1999 and 2005–2006), and Telangana (1998–1999 and 2005–2006). Tobacco prevalence for Daman & Diu was combined with Dadra & Nagar Haveli by NFHS:2019–2021. Uttarakhand, Chhattisgarh, and Jharkhand were formed in 2000 while Telangana was formed in 2014.

**Figure 3:**
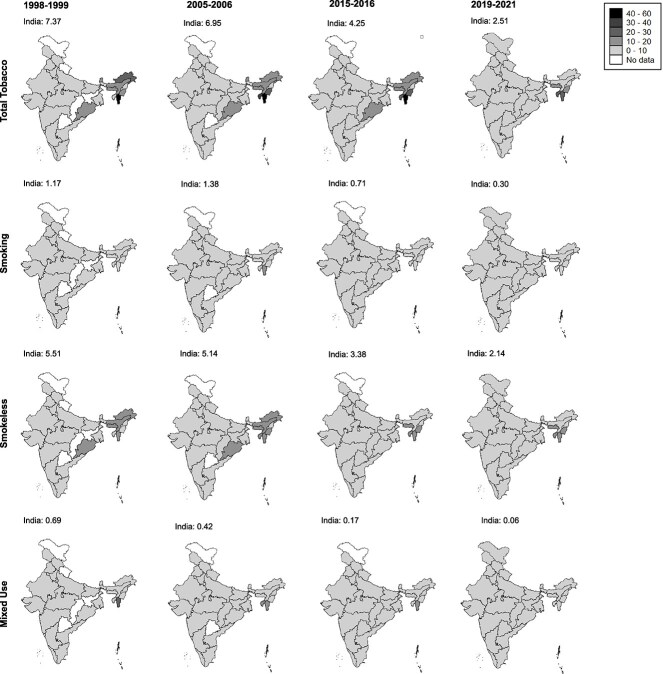
State differences in mortality fractions (%) attributable to tobacco types among women aged 35–49 years, India, 1998–2021^*^. ^*^Note: Total tobacco = sum (smoking, smokeless, and mixed use). State abbreviations: A&N = Andaman & Nicobar Islands, AP = Andhra Pradesh, ARP = Arunachal Pradesh, AS = Assam, BH = Bihar, CH = Chhattisgarh, D&N = Dadra & Nagar Haveli, D&D = Daman & Diu, DL = Delhi, GO = Goa, GJ = Gujarat, HR = Haryana, HP = Himachal Pradesh, JK = Jammu Kashmir, KA = Karnataka, KE = Kerala, LD = Ladakh, LK = Lakshadweep, MP = Madhya Pradesh, MA = Maharashtra, MN = Manipur, MG = Meghalaya, MZ = Mizoram, NG = Nagaland, OD = Odisha, PJ = Punjab, RJ = Rajasthan, SK = Sikkim, TN = Tamil Nadu, TL = Telangana, UP = Uttar Pradesh, UT = Uttarakhand, and WB = West Bengal. Prevalence of tobacco was unavailable for Uttarakhand (1998–1999), Chandigarh (1998–1999 and 2005–2006), Ladakh (1998–1999, 2005–2006, and 2015–2016), Jharkhand (1998–1999), Chhattisgarh (1998–1999), Dadra & Nagar Haveli (1998–1999 and 2005–2006), Daman & Diu (1998–1999 and 2005–2006), Lakshadweep (1998–1999 and 2005–2006), Puducherry (1998–1999 and 2005–2006), Andaman & Nicobar Islands (1998–1999 and 2005–2006), and Telangana (1998–1999 and 2005–2006). Tobacco prevalence for Daman & Diu was combined with Dadra & Nagar Haveli by NFHS:2019–2021.

Trends in mortality fractions attributable to total, smoked, smokeless, and mixed tobacco use differed by sex and state (Figures 2 and 3; Tables S1 and S2). Mortality fractions attributable to tobacco types among men (35–54 years) rose between 1998–1999 and 2005–2006 and declined thereafter. Mortality fractions attributable to total, smokeless, and mixed tobacco use declined among women (35–49 years) between 1998 and 2021, particularly from 2005–2006 onwards. Smoking-attributable mortality fractions among women increased between 1998 and 2005 and declined thereafter. Mortality fractions attributable to tobacco use were highest in 2005–2006 for men, and in 1998–1999 for women.

State differences in tobacco-attributable mortality fractions varied by tobacco-use types and sex (Figures 2 and 3), with the highest mortality fractions observed in Northeast India due to the greater mortality shares attributable to smoking among men and smokeless tobacco among women. During 2019–2021, men across 18 states in North, Central, West, East, and Northeast India, and women across 15 Indian states in West, South, East, and Northeast India, had tobacco-attributable mortality fractions significantly higher than the national average. Similar to the pattern observed for total tobacco use, mortality fractions due to smoking in 19 states and due to smokeless tobacco in 15 states were significantly higher than the national estimates. While mortality fractions attributable to tobacco types declined among most men after 2005–2006, they increased in a few North, Northeast, West, and South Indian states. More than 50% of deaths among men aged 35–54 were attributable to smoking in Meghalaya. While smoking-attributable mortality among men was highest in Northeast India, followed by North India, approximately 30% of deaths among men were attributable to smoking in South India. By contrast, mortality fractions due to smokeless tobacco use were highest in East India and were lowest in North India. Among women (35–49 years), smoking-attributable mortality fractions were highest in the Northeast Indian states, followed by the North and East Indian states, while they were lowest in the South Indian states. Similar to the pattern observed for smoking, smokeless tobacco mortality fractions among women were highest in Northeast India and in two union territories of South India. Among both men and women, mortality fractions due to mixed tobacco use were highest in the Northeast Indian states, followed by East Indian states.

## Discussion

### Summary of Results

RRs and sex differences therein differed by tobacco use type. Smoking exhibited the highest RR among men, while mixed tobacco use was highest among women. In 2019–2021, 45.7% and 2.5% of all deaths among Indian men and women, respectively, were related to tobacco use, driven by smoking-attributable mortality among men (28%), and smokeless tobacco-attributable mortality among women (2.1%). Tobacco-attributable mortality shares declined between 1998–1999 and 2019–2021, more strongly for women than men, with an increase up to 2005–2008 for the different tobacco types for men and for smoking for women. State differences in tobacco-attributable mortality shares varied by sex and tobacco types, with higher shares for smoking in the Northeast region, and for smokeless tobacco in East India.

### Explanation of Findings

Our study provides the first estimates of mortality fractions attributable to tobacco use types across Indian states over time by sex using Indian-specific prevalence data and Indian-specific RRs of dying. Applying our fractions for 2019–2021 to all-cause mortality data for the same year, 0.47 million deaths among Indian men (35–54 years) (Table S4) and 0.01 million deaths among Indian women (35–49 years) (Table S5) were estimated to relate to tobacco use. Our estimates of tobacco-attributable mortality (TAM) lie between the estimates reported by the GBD and by Kaur et al. According to the GBD estimates, approximately 0.25 million Indian men (35–54 years) and 0.004 million women (35–49 years) died due to tobacco use in India in 2021.^20^ Kaur et al. estimated 1.94 million tobacco-attributable deaths among men and 0.67 million tobacco-attributable deaths among women during 2019–2021.^6^ While both the GBD and Kaur et al. have used RRs from the American CPS study, we used Indian-specific RR estimated from the MCS to estimate TAM, which are slightly higher as compared with CPS. In addition, GBD, for recent years, relied on GATS for tobacco prevalence, which has considerable under-sampling in the top 10 tobacco-using states in India.^21^ Kaur et al., on the other hand, have estimated TAM for men and women ≥ 30 years, and included mortality attributable to second-hand smoke, whereas our study did not.^6^

Our Indian estimates of RRs of dying due to smoking (2.48; 1.44) were largely in line with those of the CPS, which estimated an RR of 2.25 among men and of 1.15 among women,^22^ as well as with estimates for other HICs^23^^,^^24^ and LMICs,^25^ and from previous Indian studies.^13^ Moreover, our RR estimates for smokeless tobacco-attributable mortality were similar to those of a previously published Indian study.^26^ In addition, the observed differences in RRs by tobacco use type were in line with those reported by the Food and Drug Administration.^27^

Our Indian RR estimates for smoking were twice as high among men as among women, while those for smokeless and mixed tobacco use were largely similar among men and women. The observed sex difference in the RR of tobacco-attributable mortality, thus, mainly stems from the sex difference in the RR for smoking-attributable mortality. This sex difference can be explained by sex differences in patterns of smoking and biological susceptibility.^28^ While men take up smoking early in life with a higher intensity, women initiate smoking at a later stage of their life with a lower frequency compared to men.^10^ On the contrary, according to the Global Adult Tobacco Survey (GATS), the average age at initiation, intensity of use, and duration of exposure for smokeless tobacco was similar for men and women,^10^ which explains the similar RR of all-cause mortality attributable to smokeless tobacco among both sexes.^28^

Our results support the general assumption that there are sex differences in tobacco-attributable mortality, with mortality fractions being higher among men than among women.^3^^,^^4^^,^^29^ In addition to the abovementioned sex differences in RRs, sex differences in prevalence, likely as a result of social factors, are important. In countries where smoking among women is socially unacceptable,^12^^,^^30^ women often use smokeless tobacco as the more acceptable alternative,^11^ which also explains the similar mortality fractions attributable to smokeless tobacco use among men and women. While smoking among women is rare due to many social factors, smokeless tobacco is quite acceptable and common across India.^12^

Our study is the first to estimate an approximately 60% elevated risk of dying due to mixed tobacco use compared to never tobacco use in India. There is evidence that mixed tobacco use among adults has been rising in 48 South-East Asian countries and is becoming a serious public health threat.^31^ Among Indian adults, mixed tobacco use rose from 6.5% in 2009–2010 to 9.8% in 2016–2017.^10^^,^^11^ Mixed tobacco users face increased risks of tobacco-related mortality, higher levels of nicotine dependence, and worse cessation rates. In light of these trends, more research on the epidemiological impact of mixed tobacco use on health is needed.^31^

Whereas among men we observed a similar trend of an increase in mortality fractions up to 2005–2006 followed by a decline for different tobacco use types, among women we observed a decline throughout the study period, except for smoking, as the pattern for smoking was the same among women and men. The observed late peak among men and women in India can be attributed to tobacco use starting later in India than in HICs. Tobacco use in India increased at a time when its risks were already well-known and control strategies were established, which led to a rapid rise and fall in mortality fractions among Indian adults. In India, men use both smoking and smokeless tobacco at a much higher rate compared to women,^10^ and are therefore exposed to long-term ill effects of tobacco, leading to higher mortality. In addition, some studies suggest that women may be more vulnerable to the harmful effects of tobacco at lower levels of exposure; however, since fewer women use tobacco, their overall disease burden remains lower than men. Moreover, women often lack social acceptance, resulting in under-reporting of tobacco use, high levels of financial dependence contributing to lower tobacco use, and higher age at initiation, which collectively contribute to a lower mortality burden.^28^ Many men, especially in LMICs, work in environments where they are exposed to additional risk factors, such as air pollution and occupational hazards, compounding the effects of smoking. Lastly, women generally have a higher life expectancy than men, which means they may survive longer and develop chronic diseases at later ages. Since tobacco-related diseases (like lung cancer and COPD) often take decades to manifest, the excess mortality in men becomes more pronounced earlier. By contrast, the different trend of mortality fractions attributable to smoking among women (similar to that among men, ie, an increase up to 2005–2006 followed by a decline) might be due to tobacco marketing in the early 20th century, which linked smoking to women’s empowerment by associating it with fashion, freedom, modern styles and values, and even weight reduction.^28^ In India, where it was not culturally correct for women to buy cigarettes openly, tobacco companies offered to deliver them to women at their doorsteps,^28^ which might have led to an increase in smoking prevalence among women during the first half of the 2000s. However, as tobacco awareness and control policies for smoking increased in India during the late 2000s, smoking prevalence declined among both women and men, which in turn led to declines in smoking-attributable mortality fractions.

Our results also highlight important state differences in TAMF, which, interestingly, differed not only by sex but also by tobacco use type. Northeast states had the highest mortality fractions due to tobacco use among both men and women, which were driven by high smoking-attributable mortality fractions reflecting higher underlying intensities and duration of tobacco use and smoking.^12^ This can be explained by greater cultural and societal acceptance of tobacco use in these states, more access to and availability of tobacco products, less effective implementation of state policies and lower levels of adherence to these policies, increased female employment in the tobacco sector, and higher levels of illicit trading of tobacco products.^12^^,^^32^ Higher mortality fractions attributable to smokeless tobacco in East India among men may be explained by a combination of cultural, social, economic, and regulatory factors.^33^ For example, in Bihar, use of smokeless tobacco is deeply ingrained in their local culture, and has been passed down through generations as a normal part of daily life. In addition, given that a significant portion of the population in Bihar lives below the poverty line, affordability of smokeless tobacco makes it more accessible, and tobacco companies have aggressively marketed smokeless tobacco products there, often targeting younger populations and lower-income groups.^33^

### Strengths and Limitations of the Study

We used detailed prevalence data (by sex and state) obtained from the large and representative population-based NFHS data. We used the NFHS instead of the GATS because the GATS has considerable under-sampling in the top 10 tobacco-using states,^21^ which would likely result in considerable underestimation of mortality fractions attributable to tobacco use types. Moreover, the GATS did not go far back in time, as the first survey was conducted in 2009–2010, and thus six years after India implemented the Cigarettes and Other Tobacco Products Act (COTPA) in 2003, 5 years after ratification of the WHO Framework Convention on Tobacco Control (FCTC) in 2004, and two years after India implemented the National Tobacco Control Policy (NTCP) in 2007–2008. An important downside of using NFHS is that it provides tobacco use information only for men aged 35–54 and women aged 35–49. See below as well.

We used a direct prevalence-based approach instead of an indirect method (like the Peto-Lopez method and the Preston–Glei–Wilmoth method).^34^ We did so because the latter methods assume that lung cancer is primarily caused by tobacco smoking, and that lung cancer mortality is very low among non-smokers,^35^ which is not the case for many LMICs like India due to environmental factors such as air pollution, passive smoking, and cooking fuel interacting with smoking. A general downside of using the direct prevalence-based approach is, however, that the resulting estimates rely purely on tobacco prevalence, and not on aspects such as tobacco use intensity and duration of use.^36^ In addition, because of a time lag between tobacco use and tobacco-attributable mortality of about 20–30 years, depending on the context,^3^ estimated fractions based on tobacco prevalence will only apply to the mortality situation several years later. However, no large effect is expected for the observed differences between the different tobacco use types, between the sexes, between the states, and for different years.

We applied RRs for broad age groups (35–49 years for women and 35–54 years for men) to estimate mortality fractions attributable to tobacco use, because reliable age-specific RRs, particularly for smoking among women by 5-year age groups could not be estimated based on the MCS, and because the NFHS provided tobacco use data only for the abovementioned age groups. While using RRs for broad age groups instead of for 5-year age groups likely resulted in slightly biased estimates, the effect is difficult to assess. Moreover, our estimates apply to 35- to 49-year-old women and 35- to 54-year-old men, and should not be interpreted as mortality fractions attributable to tobacco use for Indian men and women aged 35 and over. Whereas tobacco use prevalence tends to be highest at ages 35–49 and 35–54,^10^ RRs of dying from tobacco use tend to be highest around ages 50–69 (particularly among women), and decline thereafter,^37^ in line with the many other potential causes of death at older ages.

We used Indian sex-specific RRs by tobacco use type (smoked, smokeless, and mixed tobacco use), based on data from the MCS. Although the MCS was conducted only in the Mumbai metropolitan area, which might not be representative of the general Indian population, the RRs of mortality due to smoking and smokeless tobacco use from the MCS were similar to those estimated from the MDS^13^ and other pooled meta-analysis studies conducted in India,^26^ and were therefore considered sufficiently reliable to be applied to the present study. We used the MCS to estimate RRs, as other Indian studies estimated RRs only due to smoking, and detailed data were not available.

To obtain comparable RRs across Indian states and over time, we applied a time-invariant and non-state-specific set of RRs, because large prospective cohort studies analyzing tobacco harms across time in India and states are missing.^3^^,^^35^ Previous studies comparing trends in smoking-attributable mortality between countries have generally used the same approach.^38^ An important implication of this approach is, however, that the observed trends and geographic differences in tobacco-attributable mortality fractions purely reflect the differences in prevalence, and not state differences and secular trends in the treatment of tobacco use-related diseases. Our estimates of tobacco-attributable mortality rely on standard epidemiological methods that assume a causal link between tobacco use and mortality. These estimates reflect the proportion of deaths that could potentially be avoided if tobacco use were eliminated, based on prior research linking tobacco exposure to increased mortality risk. However, as these calculations rely on observational data, they should be interpreted as an estimate of public health impact rather than direct proof of causation.

## Conclusions and Implications

Levels, sex differences, regional differences, and time trends in the mortality fractions attributable to tobacco use in India differ substantially by tobacco use type. Our findings have important implications for policy-making and society. The Government of India has taken substantial steps to strengthen tobacco control by implementing the COTPA in 2003, WHO-FCTC in 2004, and NTCP in 2007–2008.^20^ However, effective implementation of these measures varies significantly across Indian states. Our study’s findings highlight the importance of further strengthening tobacco control initiatives for India by shifting to a target-oriented approach comprising different actions for each tobacco use type and specifically aimed at men and the Northeast Indian states, to enable India to achieve its Sustainable Development Goals (SDG) by 2030.

For Indian society, investments in public awareness of the impact of all types of tobacco use on human health are of utmost importance, as there is no safe level of exposure to tobacco, and tobacco use in any form is harmful to the body.^2^

## Supplementary Material

Manuscript_Clean_Copy_ntaf121

Supplementary_Material_File1_Data_and_Method_ntaf121

Supplementary_Material_File2_Supplementary_Tables_ntaf121

## Data Availability

The NFHS data are publicly available from the DHS website. The Mumbai Cohort Study data will be available upon request after completing due data request formalities of the Healis.
