# Supplementary material for: Mortality Fractions Attributable to Smoking and Smokeless and Mixed Tobacco Use Among Men and Women Across India, 1998–2021
Source: Nicotine Tob Res. 2025 Jun 14;27(12):2247–55. doi: 10.1093/ntr/ntaf121 (PMC12641177; doi:10.1093/ntr/ntaf121)
Supplement: Supplementary_Material_File1_Data_and_Method_ntaf121 [file supplementary_material_file1_data_and_method_ntaf121.docx]

**Supplementary Material: Data and Methods**

**Mortality Fractions Attributable to Smoking, Smokeless and Mixed Tobacco Use among Men and Women across India, 1998-2021.**

**Table of Contents**

[**Estimating Tobacco Prevalence** 2](#_Toc190774547)

[**Estimation of Relative Risk (RR)** 3](#_Toc190774548)

[**Estimation of Tobacco Attributable Mortality Fractions (TAMF)** 5](#_Toc190774549)

[**Data Control Process** 7](#_Toc190774550)

[**References** 9](#_Toc190774551)

## **Estimating Tobacco Prevalence**

We use data from second, third, fourth and fifth rounds (1998–2021) of the National Family Health Survey (NFHS) to estimate national sand state-level tobacco prevalence for men (15-54 years) and women (15–49 years). The NFHS is a nationally representative, cross- sectional household and individual sample survey that represents 99% of the Indian population living in 37 states/union territories [[33](#_bookmark31)–[35](#_bookmark32)]. It is the only population survey in the country that provides reliable estimates for various socio-demographic, lifestyle and morbidity indicators, for females aged 15–49 and males aged 15–54. Information on tobacco-use of household members was collected through self-administered questionnaires that included the following four questions: 1) “Do you currently chew pan masala or tobacco?” “Do you currently smoke cigarettes or bidis?” “Do you currently smoke or use tobacco in any other form?” “In what other forms do you currently smoke or use tobacco?” These questions were answered by the head of the household in the 1998-99 round, and by each individual respondent in the other two rounds. We categorized individuals as “tobacco users” if the respondent answered “yes” to either one of the first three questions, and thus combined tobacco smoking and smokeless tobacco use. In addition, we distinguished between tobacco smoking (smoking of cigarettes, bidis, pipes/hookah, and other items), smokeless tobacco use (consumption of products like ghutka, pan masala, snuff, and khaini), and mixed-use tobacco (consumption of smoked and smokeless tobacco). We defined individuals as smokers if they only smoked cigarettes or bidis, and did not use any other form of chewing tobacco. Smokeless tobacco users were defined as those who consumed tobacco orally and had never used smoked tobacco. Lastly, individuals who consumed both smoked and smokeless tobacco were classified as mixed-tobacco users.

We created mutually exclusive groups for tobacco types, and calculated the prevalence of tobacco smoking, smokeless tobacco and mixed-use tobacco for men (15-54 years) and women (15–49 years) for the years 1998-99, 2005-06, 2015-16 and 2019-2021 for India and 37 states/union-territories. For the estimation of representative national and sub-national age-specific tobacco use prevalence, we applied appropriate sampling weights that account for the multi- stage cluster sampling design of the NFHS. The sampling weights were calculated by the NFHS based on sampling probabilities separately for each sampling stage and for each cluster.

The sampling weight for each household in cluster *i* of stratum *h* is the inverse of its overall selection probabil- ity: W*_hi_* = 1/P*_hi_*.

Where, P*_hi_* is the second stage sampling probability within the ith cluster.

The household sampling weight was further adjusted by the NFHS for individual non-response to obtain individual sampling weight separately for men and women. These sampling weights were normalized at the national level to obtain national standard weights and at the state level to obtain state standard weights. We multiplied the state standard weights with tobacco percentage to obtain age-specific tobacco prevalence for men and women.

## **Estimation of Relative Risk (RR)**

We used the Mumbai Cohort Study (MCS) to estimate the sex specific relative risk (RR) attributable to tobacco types. A total of 148,173 individuals aged ≥35 years were recruited during 1991–97 in the

cohort. The voters’ lists were used as the sampling frame. These lists provided details including name, age, sex and address of all adults and were grouped into polling stations comprising 1000–1500 voters. The baseline survey included the following 2 components: (1) anthropometric measurements by weight and height and (2) interviewer administration of a structured questionnaire to obtained information on age, gender, education, religion, mother tongue (language), and information related to frequency and type of tobacco usage. An active house-to-house follow-up was conducted 5–6 years after the initial survey. The field investigators were provided with the list of names and addresses of the cohort members and instructed to revisit each person. A personal interview was conducted if the participant was alive and available. In the event of death of the participant, the date and place of death were recorded with maximal accuracy. Permanent migration from the study area was considered as withdrawal from the study, and the date of migration was noted. The re-interviews were conducted between 1997 and 2003.

Under the uniform system of registration of births and deaths, reporting and registration of births and deaths is compulsory in India. Although the quality of registration varies across different parts of the country, in Mumbai almost all the deaths are registered and certified medically. The cause of death was sought not from household members but from the municipal corporation death records. For all deaths over 27 years of age in those records, details (including demographic information on the decedent, and the underlying cause(s) of death) were computerized by project personnel— amounting to over 20 000 deaths per year within the study area. The deaths recorded during the follow-up of the cohort were matched with the dataset from the corporation. The matching was done using age, sex, name, address, and date of death. The most important variables for matching were name and address, since the spelling could be very different in two datasets most of the matching was done manually. For 1685 matched deaths an independent trained field supervisor was sent to the household. In all the 1685 revisited deaths matching was found to be 100% correct.

Respondents were interviewed and classified according to present and past tobacco use as (a) having never used tobacco, (b) ex-smoker, (c) ex-smokeless tobacco user, (d) ever smoker, (f) ever smokeless tobacco user, and (g) ever mixed user (smokeless and smoking). We combined ex-smoker and ever-smoker, ever-smokeless and ex-smokeless tobacco together to estimate prevalence of smoking and smokeless tobacco. Person-years were calculated using the date of recruitment and the date of withdrawal (defined as the date of death; re- interview; migration; or ascertainment). In cases where the exact date of death (2.6% deaths) or migration (1.4% migrations) was not available, mid-point between date of recruitment and date of ascertainment was used. The information on age, sex, and details of tobacco use was abstracted from the baseline data. Direct age-adjustment was done using overall 5-year age-specific person-years as weights. Multivariate weighted analysis was performed by using Cox proportional hazard regression modelling (Cox 1972), controlling for education (categorized as illiterate, primary, middle, secondary, and college level) as a proxy for socio-economic status was applied and adjusted sex-specific RRs with 95% CIs were estimated for tobacco smoking, smokeless ad mixed-use tobacco. We used the Schoenfeld Test to assess the proportional hazards assumption (Table 1a and Table 1b). The response variable, death, was a dichotomous variable (yes or no) and time to event (or censor) was continuous.

Table1a: Schoenfeld Test to assess the Proportional Hazard (PH) assumption for men.

| **Time function: Analysis time** | | | | | |
| --- | --- | --- | --- | --- | --- |
| **Variable** | | **rho** | **chi2** | **df** | **Prob>chi2** |
| Tobacco Use | Non-users |  | . | 1 | . |
|  | Smokers | -0.00396 | 0.15 | 1 | 0.6988 |
|  | Smokeless | 0.00965 | 0.88 | 1 | 0.3473 |
|  | Mixed-use | -0.00051 | 0 | 1 | 0.9603 |
| Age | | -0.0082 | 0.59 | 1 | 0.4414 |
| Education | | 0.0271 | 6.63 | 1 | 0.11 |
|  | | | | | |
| **Global test** | |  | **8.89** | **5** | **0.1133** |

Table1b: Schoenfeld Test to assess the Proportional Hazard (PH) assumption for women.

| **Time function: Analysis time** | | | | | |
| --- | --- | --- | --- | --- | --- |
| **Variable** | | **rho** | **chi2** | **df** | **Prob>chi2** |
| Tobacco Use | Non-users | . | . | 1 | . |
|  | Smokers | 0.0442 | 6.84 | 1 | 0.5789 |
|  | Smokeless | 0.01627 | 0.9 | 1 | 0.3419 |
|  | Mixed-use | 0.05264 | 9.44 | 1 | 0.2321 |
| Age | | -0.0043 | 0.06 | 1 | 0.8123 |
| Education | | -0.0028 | 0.03 | 1 | 0.862 |
|  | | | | | |
| **Global test** | |  | **15.62** | **5** | **0.108** |

The Cox Proportional Hazard Model produces relative hazard ratios (HR), and not absolute hazard ratios to assess the association between tobacco use types and death. Under situations, where the proportional hazard assumptions are met and time to follow-up is small (in our case 5 years since), the relative hazard ratio as obtained from the Cox-Proportional Hazard model can be approximated to Relative Risks (RR)(1).

Additionally, since the MCS was confined to only Mumbai metropolitan city, we could not use mixed-effects model to incorporate territorial variation. Therefore, we restricted ourself to the widely used cox-proportional hazard model to estimate the relative risk of all-cause mortality attributable to tobacco types.

## **Estimation of Tobacco Attributable Mortality Fractions (TAMF)**

Population-attributable fractions (equation1) were calculated to obtain sex-specific mortality fractions attributable to tobacco smoking, smokeless tobacco and mixed-use tobacco for men (35-54 years) and women (35-49 years).

TAMF_s,c,t,y_ = p_s,c,t,y_ (RR_s,t_-1) / [1 + (p_s,c,t,y_ (RR_s,t_-1))] (1)

where:

TAMF represents the tobacco-attributable mortality fraction, estimated separately for:

1. Men (ages 35-54) and Women (ages 35-49)
2. p denotes the prevalence of tobacco consumption, disaggregated by:

- Tobacco-use type (smoked, smokeless, and mixed tobacco-use)

1. Sex (men and women)
2. Context (India as a whole or one of the 37 individual states/union territories)
3. Survey year (1998-99, 2005-06, 2015-16, and 2019-21)

RR refers to the relative risk (RR) of all-cause mortality for tobacco users compared to a baseline of non-users. These estimates were derived from the MCS, with RR values varying based on tobacco-use type (smoking, smokeless, or mixed use).

We computed **TAMF values separately for each sex, tobacco type, and Indian state/union territory** for all four time periods under study. To estimate the total mortality fraction attributable to tobacco use in each population, we **summed up the individual TAMFs for each tobacco-use category (smoked, smokeless, and mixed-use)**. This step was necessary because the **RR values derived from the MCS reflect the combined impact of all tobacco-use types in proportion to their prevalence**.

Given that **cause-specific mortality data varied in completeness and quality across Indian states and sexes**, we estimated **tobacco-attributable mortality fractions for all-cause mortality**, rather than for specific causes of death. This approach is consistent with several prior studies on tobacco-attributable mortality in India (19,20).

Additionally, as the MCS data is restricted to Mumbai metropolitan city and uses education as a proxy for socio-economic status, incorporating territorial variation through a mixed-effect model was not feasible. Therefore, we restricted ourself to the widely used cox-proportional hazard model to estimate the relative risk of mortality attributable to tobacco types*.*

# **Data Control Process**

NFHS collects information on a wide variety of indicators that assist policymakers and programme managers to formulate, implement and monitor programmes and strategies to achieve health, family welfare, and other development goals. The nodal agency (IIPS, Mumbai) is responsible for developing and finalizing the questionnaires based on advice received from key stakeholders and the Technical Advisory Committee, and also on the lessons learned from the pretest of all the questionnaires. To ensure that all persons involved in the NFHS survey understand the field protocols and the contents of the instruments, particularly the field investigators and supervisors, various manuals are prepared (Interviewer’s Manual, Supervisor’s Manual, Health Investigator’s Manual, Household Mapping and List Manual and other manuals) are prepared. Also, fieldwork related forms, referral letters, information pamphlets, etc. are prepared for distribution during fieldwork. All survey instruments and field procedures are pretested in the field at a suitable location. Interviewers, supervisors and health investigators who will be part of the pretest exercise are trained to implement the questionnaires and field procedures, including the clinical, anthropometric and biochemical (CAB) component. Feedback from the pretest exercise is used to finalize the questionnaires in English and Hindi. Accordingly, all the manuals are also revised and finalized for use during the main survey fieldwork. Various training of trainers (ToT) workshops are held to provide training to trainers for the different aspects of the survey. The trained FA staff then provide training to the field staff recruited by them to carry out fieldwork in the states assigned to them. The training is guided by the standard survey documents to ensure that the same rigorous procedures are followed throughout the country. At the state level, in-depth training is typically conducted for at least four weeks, and only trainees that pass a test that is graded by IIPS are retained for conducting fieldwork.

Different protocols for mapping and listing of households, training of field staff, team supervisor, CAB Team, Information Technology (IT) personals, and assessment of non-sampling errors have been developed for rigorous data assurance and data quality checks at various stages of data collection in NFHS. More information on the data control measures have been described elsewhere.(2)

The Mumbai Cohort Study (MCS) used careful mapping to ascertain the target population and an active house-to-house method for enrolling subjects and determining their vital status at the time of follow-up. This method provided accurate information for >95% of the original cohort that was followed-up. The MCS excluded polling stations comprising upper– middle class and upper-class housing complexes that were not accessible due to security issues. Similarly, the study excluded homeless persons, such as footpath dwellers, as they are generally excluded from the voters’ list. The electoral rolls were used as the selection frame for individuals. These rolls provided name, age, sex, and address of all individuals aged 18 years and above. The rolls er assumed to be complete as almost everyone is entitled to vote. Rolls are updated before every major election through house-to-house visits. All eligible people (age 35 years and older) listed in the electoral roll were approached by investigators and their residence status was confirmed through their listing in “ration cards”, that are issues by the Bombay Municipal Corporation. The interviews were conducted by trained investigators using handled computers, that provided appropriate sequencing of questions, skipped irrelevant questions, accepted only valid codes for answers, and performed range and consistency checks on the spot.

Under the uniform system of registration of births and deaths, reporting and registration of births and deaths is compulsory in India. Although the quality of registration varies across different parts of the country, in Mumbai almost all the deaths are registered and certified medically. In Mumbai, death is reported on a standard death certificate in accordance with the WHO guidelines. Considering the diagnosis of ill-defined causes as a marker for the quality of causes of death certification, in Mumbai Cohort Study the proportion of ill-defined causes among matched deaths were <5% [International Classification of Diseases (ICD) codes R50-69, R95-99]. For all deaths that occurred with the MCS area, additional details including demographic information of the decedent and the underlying cause(s) of death were recorded from BMC death registers by project personnel. This work amounted to record approximately over 20,000 deaths per year from BMC death registers. Then the deaths recorded during the follow-up of the cohort were linked with the dataset obtained from BMC death registers. Linking was performed using age, sex, name, address and date of expiry. The most important variables for linking were found to be name and address of the deceased. Linking was performed manually in order to obviate any errors due to the differences in the spelling of names between the two datasets. For matched deaths, the underlying cause of death was derived from the cause of information coped from the corporation death registers and was coded according to the ICD-10 guidelines. Also for 1685 randomly selected matched deaths, an independent check was performed by sending trained field investigator to the household of the deceased. In all the 1685 revisited deaths, matching was found to be 100% accurate. More information on the MCS can be found elsewhere(3).

# **References**

1. Hernán MA. The hazards of hazard ratios. Vol. 21, Epidemiology. 2010. p. 13–5.

2. International Institute for Population Sciences (IIPS). National Family Health Survey 2019-2021: Data Quality Assurance and Quality Control Mechanism. 2021.

3. Gupta PC, Pednekar MS, Parkin DM, Sankaranarayanan R. Tobacco associated mortality in Mumbai (Bombay) India. Results of the Bombay Cohort Study. Int J Epidemiol. 2005 Dec;34(6):1395–402.
