# Supplementary material for: Mortality Fractions Attributable to Smoking and Smokeless and Mixed Tobacco Use Among Men and Women Across India, 1998–2021
Source: Nicotine Tob Res. 2025 Jun 14;27(12):2247–55. doi: 10.1093/ntr/ntaf121 (PMC12641177; doi:10.1093/ntr/ntaf121)
Supplement: Supplementary_Material_File2_Supplementary_Tables_ntaf121 [file supplementary_material_file2_supplementary_tables_ntaf121.docx]

**Supplementary Material: Result**

**Mortality Fractions Attributable to Smoking, Smokeless and Mixed Tobacco Use among Men and Women across India, 1998-2021.**

**Table of Contents**

[**Table S1: Age-sex distribution of India during 2021 according to the World Population Prospects.** 2](#_Toc190157160)

[**Table S2: Mortality Fractions (%) Attributable to smoking, smokeless and mixed tobacco use among men aged 35-54 years across 37 Indian states from 1998-1999 to 2019-2021.** 2](#_Toc190157161)

[**Table S3: Mortality Fractions (%) Attributable to smoking, smokeless and mixed tobacco use among women aged 35-49 years across 37 Indian states from 1998-1999 to 2019-2021.** 6](#_Toc190157162)

[**Table S4: Deaths (in million) attributable to tobacco use type among men (35-54 years) in India across states during 2019-2021.** 10](#_Toc190157163)

[**Table S5: Deaths (in million) attributable to tobacco use type among women (35-49 years) in India across states during 2019-2021.** 11](#_Toc190157164)

##

## **Table S1: Age-sex distribution of India during 2021 according to the World Population Prospects.**

| **Age Group** | **Population (n^'000^(%))** | |
| --- | --- | --- |
|  | **Male** | **Female** |
| 0-4 | 60890.05 (8.34) | 56195.13 (8.21) |
| 5-9 | 63938.17 (8.76) | 58444.80 (8.54) |
| 10-14 | 66447.88 (9.10) | 60435.77 (8.33) |
| 15-19 | 68378.66 (9.37) | 62098.79 (9.08) |
| 20-24 | 67118.72 (9.20) | 60799.12 (8.89) |
| 25-29 | 63786.67 (8.74) | 58239.31 (8.51) |
| 30-34 | 60236.18 (8.25) | 55548.77 (8.12) |
| 35-39 | 55044.61 (7.54) | 50846.89 (7.43) |
| 40-44 | 47879.39 (6.56) | 44646.29 (6.52) |
| 45-49 | 41599.13 (5.70) | 39534.51 (5.78) |
| 50-54 | 35642.93 (4.88) | 34428.14 (5.03) |
| 55-59 | 30308.03 (4.15) | 29622.42 (4.33) |
| 60-64 | 24812.28 (3.40) | 24710.45 (3.61) |
| 65-69 | 18914.09 (2.59) | 19446.70 (2.84) |
| 70-74 | 11997.32 (1.64) | 13026.20 (1.90) |
| 75-79 | 6883.34 (0.94) | 8135.68 (01.19) |
| 80-84 | 3786.01 (0.52) | 4905.96 (0.72) |
| 85-89 | 1650.16 (0.23) | 2320.45 (0.34) |
| 90-94 | 506.41 (0.07) | 735.32 (0.11) |
| 95-99 | 94.42 (0.01) | 143.24 (0.02) |
| 100+ | 9.6585 (0.001) | 15.87 (0.002) |

Source: World Population Prospects

## **Table S2: Mortality Fractions (%) Attributable to smoking, smokeless and mixed tobacco use among men aged 35-54 years across 37 Indian states from 1998-1999 to 2019-2021.**

| **State** | **Year** | **Total Tobacco** | **Smoking** | **Smokeless** | **Mixed-Use** |
| --- | --- | --- | --- | --- | --- |
| **India** | 1998-1999 | 56.70 | 33.04 | 7.52 | 16.15 |
|  | 2005-2006 | 67.97 | 39.52 | 11.80 | 16.65 |
|  | 2015-2016 | 54.32 | 32.86 | 10.51 | 10.95 |
|  | 2019-2021 | 45.67 | 28.03 | 9.61 | 8.03 |
| **Jammu Kashmir** | 1998-1999 | 57.64 | 46.88 | 0.95 | 9.81 |
|  | 2005-2006 | 47.67 | 40.59 | 3.64 | 3.44 |
|  | 2015-2016 | 48.65 | 41.29 | 2.76 | 4.60 |
|  | 2019-2021 | 45.36 | 39.59 | 1.64 | 4.13 |
| **Himachal Pradesh** | 1998-1999 | 54.24 | 43.89 | 1.22 | 9.13 |
|  | 2005-2006 | 49.77 | 41.52 | 3.72 | 4.53 |
|  | 2015-2016 | 51.29 | 42.21 | 3.42 | 5.66 |
|  | 2019-2021 | 49.47 | 40.40 | 3.13 | 5.93 |
| **Punjab** | 1998-1999 | 27.26 | 20.33 | 3.17 | 3.76 |
|  | 2005-2006 | 35.90 | 26.86 | 5.61 | 3.43 |
|  | 2015-2016 | 28.23 | 21.33 | 3.79 | 3.11 |
|  | 2019-2021 | 26.75 | 20.75 | 2.87 | 3.13 |
| **Chandigarh** | 1998-1999 |  |  |  |  |
|  | 2005-2006 |  |  |  |  |
|  | 2015-2016 | 35.75 | 25.61 | 3.53 | 6.60 |
|  | 2019-2021 | 35.92 | 26.39 | 4.76 | 4.77 |
| **Uttarakhand** | 1998-1999 |  |  |  |  |
|  | 2005-2006 | 73.78 | 44.22 | 10.57 | 18.99 |
|  | 2015-2016 | 60.73 | 43.09 | 6.67 | 10.97 |
|  | 2019-2021 | 62.18 | 36.67 | 10.53 | 14.98 |
| **Haryana** | 1998-1999 | 55.70 | 45.09 | 1.30 | 9.31 |
|  | 2005-2006 | 53.73 | 47.88 | 2.38 | 3.47 |
|  | 2015-2016 | 45.45 | 41.11 | 2.07 | 2.27 |
|  | 2019-2021 | 41.70 | 37.43 | 1.85 | 2.42 |
| **Delhi** | 1998-1999 | 42.99 | 32.55 | 2.85 | 7.59 |
|  | 2005-2006 | 53.27 | 37.79 | 6.66 | 8.82 |
|  | 2015-2016 | 43.14 | 31.83 | 6.29 | 5.03 |
|  | 2019-2021 | 47.67 | 33.08 | 6.46 | 8.13 |
| **Rajasthan** | 1998-1999 | 61.12 | 43.00 | 3.92 | 14.20 |
|  | 2005-2006 | 71.03 | 46.67 | 8.12 | 16.24 |
|  | 2015-2016 | 60.27 | 38.67 | 9.34 | 12.25 |
|  | 2019-2021 | 60.36 | 37.36 | 9.85 | 13.14 |
| **Uttar Pradesh** | 1998-1999 | 66.35 | 33.84 | 8.95 | 23.56 |
|  | 2005-2006 | 84.56 | 43.57 | 15.20 | 25.78 |
|  | 2015-2016 | 73.58 | 37.22 | 15.28 | 21.08 |
|  | 2019-2021 | 62.47 | 31.37 | 14.54 | 16.55 |
| **Ladakh** | 1998-1999 |  |  |  |  |
|  | 2005-2006 |  |  |  |  |
|  | 2015-2016 |  |  |  |  |
|  | 2019-2021 | 39.89 | 32.03 | 2.33 | 5.53 |
| **Bihar** | 1998-1999 | 53.09 | 11.20 | 15.90 | 25.98 |
|  | 2005-2006 | 80.15 | 33.55 | 20.02 | 26.57 |
|  | 2015-2016 | 61.39 | 25.68 | 17.61 | 18.09 |
|  | 2019-2021 | 54.25 | 20.51 | 18.27 | 15.47 |
| **West Bengal** | 1998-1999 | 65.52 | 42.40 | 5.42 | 17.70 |
|  | 2005-2006 | 79.00 | 47.23 | 10.54 | 21.23 |
|  | 2015-2016 | 67.17 | 42.49 | 9.77 | 14.91 |
|  | 2019-2021 | 63.78 | 42.51 | 8.14 | 13.14 |
| **Jharkhand** | 1998-1999 |  |  |  |  |
|  | 2005-2006 | 63.62 | 25.46 | 18.84 | 19.31 |
|  | 2015-2016 | 47.77 | 19.21 | 16.23 | 12.34 |
|  | 2019-2021 | 51.63 | 19.49 | 17.67 | 14.48 |
| **Odisha** | 1998-1999 | 53.56 | 15.78 | 14.21 | 23.56 |
|  | 2005-2006 | 81.53 | 35.69 | 19.35 | 26.49 |
|  | 2015-2016 | 66.21 | 28.88 | 17.55 | 19.79 |
|  | 2019-2021 | 46.35 | 22.04 | 14.00 | 10.30 |
| **Sikkim** | 1998-1999 | 44.21 | 15.55 | 13.68 | 14.97 |
|  | 2005-2006 | 60.57 | 29.72 | 15.49 | 15.35 |
|  | 2015-2016 | 34.13 | 22.71 | 6.80 | 4.62 |
|  | 2019-2021 | 38.19 | 24.77 | 7.37 | 6.05 |
| **Arunachal Pradesh** | 1998-1999 | 62.67 | 17.67 | 18.00 | 27.00 |
|  | 2005-2006 | 61.43 | 28.07 | 16.39 | 16.98 |
|  | 2015-2016 | 70.85 | 36.75 | 14.13 | 19.96 |
|  | 2019-2021 | 67.07 | 36.64 | 12.35 | 18.07 |
| **Nagaland** | 1998-1999 | 57.36 | 22.56 | 9.50 | 25.31 |
|  | 2005-2006 | 76.67 | 38.15 | 15.77 | 22.76 |
|  | 2015-2016 | 72.67 | 34.56 | 16.44 | 21.68 |
|  | 2019-2021 | 58.95 | 31.37 | 12.63 | 14.95 |
| **Manipur** | 1998-1999 | 65.09 | 34.85 | 8.15 | 22.08 |
|  | 2005-2006 | 84.04 | 40.57 | 17.17 | 26.30 |
|  | 2015-2016 | 87.45 | 40.75 | 18.54 | 28.15 |
|  | 2019-2021 | 76.46 | 38.46 | 15.42 | 22.59 |
| **Mizoram** | 1998-1999 | 82.47 | 35.94 | 12.17 | 34.37 |
|  | 2005-2006 | 94.73 | 50.12 | 14.50 | 30.11 |
|  | 2015-2016 | 100.00 | 51.88 | 16.02 | 32.79 |
|  | 2019-2021 | 88.91 | 52.92 | 9.42 | 26.57 |
| **Tripura** | 1998-1999 | 66.01 | 50.95 | 0.88 | 14.18 |
|  | 2005-2006 | 97.54 | 51.76 | 14.25 | 31.53 |
|  | 2015-2016 | 89.23 | 49.66 | 12.38 | 27.19 |
|  | 2019-2021 | 66.53 | 45.13 | 7.35 | 14.05 |
| **Meghalaya** | 1998-1999 | 73.34 | 49.33 | 3.29 | 20.72 |
|  | 2005-2006 | 94.96 | 51.76 | 13.49 | 29.71 |
|  | 2015-2016 | 85.59 | 51.73 | 9.05 | 24.82 |
|  | 2019-2021 | 67.93 | 48.73 | 4.69 | 14.50 |
| **Assam** | 1998-1999 | 67.19 | 24.94 | 14.75 | 27.51 |
|  | 2005-2006 | 78.53 | 35.31 | 18.35 | 24.87 |
|  | 2015-2016 | 72.64 | 35.40 | 16.04 | 21.20 |
|  | 2019-2021 | 62.53 | 33.45 | 13.28 | 15.80 |
| **Chhattisgarh** | 1998-1999 |  |  |  |  |
|  | 2005-2006 | 69.37 | 36.77 | 14.09 | 18.52 |
|  | 2015-2016 | 51.85 | 25.18 | 14.27 | 12.40 |
|  | 2019-2021 | 52.13 | 28.02 | 11.84 | 12.26 |
| **Madhya Pradesh** | 1998-1999 | 63.19 | 28.92 | 11.78 | 22.48 |
|  | 2005-2006 | 84.45 | 43.71 | 15.07 | 25.68 |
|  | 2015-2016 | 72.08 | 37.69 | 14.45 | 19.94 |
|  | 2019-2021 | 54.58 | 29.12 | 13.17 | 12.29 |
| **Gujarat** | 1998-1999 | 46.72 | 31.95 | 5.98 | 8.80 |
|  | 2005-2006 | 63.04 | 36.35 | 12.14 | 14.55 |
|  | 2015-2016 | 53.60 | 27.40 | 14.41 | 11.79 |
|  | 2019-2021 | 39.37 | 19.52 | 13.20 | 6.65 |
| **Dadra &  Nagar Haveli** | 1998-1999 |  |  |  |  |
|  | 2005-2006 |  |  |  |  |
|  | 2015-2016 | 46.35 | 26.59 | 10.45 | 9.31 |
|  | 2019-2021 | 47.75 | 21.95 | 13.37 | 12.43 |
| **Daman & Diu** | 1998-1999 |  |  |  |  |
|  | 2005-2006 |  |  |  |  |
|  | 2015-2016 | 31.68 | 19.14 | 8.74 | 3.80 |
|  | 2019-2021 |  |  |  |  |
| **Maharashtra** | 1998-1999 | 40.02 | 18.22 | 11.81 | 9.98 |
|  | 2005-2006 | 51.87 | 27.60 | 13.60 | 10.67 |
|  | 2015-2016 | 34.20 | 17.48 | 11.86 | 4.86 |
|  | 2019-2021 | 29.98 | 14.94 | 10.56 | 4.48 |
| **Andhra Pradesh** | 1998-1999 | 52.85 | 41.92 | 1.98 | 8.95 |
|  | 2005-2006 | 52.71 | 42.46 | 4.07 | 6.19 |
|  | 2015-2016 | 37.07 | 33.02 | 2.38 | 1.67 |
|  | 2019-2021 | 34.36 | 29.35 | 2.66 | 2.35 |
| **Karnataka** | 1998-1999 | 45.84 | 35.33 | 3.55 | 6.96 |
|  | 2005-2006 | 50.40 | 37.19 | 6.74 | 6.46 |
|  | 2015-2016 | 42.44 | 31.19 | 5.86 | 5.40 |
|  | 2019-2021 | 31.76 | 23.68 | 4.64 | 3.44 |
| **Goa** | 1998-1999 | 33.43 | 28.05 | 2.06 | 3.31 |
|  | 2005-2006 | 30.18 | 23.44 | 4.74 | 2.00 |
|  | 2015-2016 | 22.07 | 15.95 | 3.83 | 2.29 |
|  | 2019-2021 | 29.96 | 21.56 | 4.98 | 3.41 |
| **Lakshadweep** | 1998-1999 |  |  |  |  |
|  | 2005-2006 |  |  |  |  |
|  | 2015-2016 | 38.10 | 34.44 | 1.51 | 2.14 |
|  | 2019-2021 | 46.84 | 32.30 | 6.53 | 8.01 |
| **Kerala** | 1998-1999 | 49.38 | 39.55 | 1.81 | 8.02 |
|  | 2005-2006 | 52.27 | 39.63 | 4.73 | 7.92 |
|  | 2015-2016 | 34.12 | 29.95 | 1.97 | 2.20 |
|  | 2019-2021 | 29.23 | 24.58 | 1.64 | 3.02 |
| **Tamil Nadu** | 1998-1999 | 43.70 | 34.76 | 3.03 | 5.92 |
|  | 2005-2006 | 49.68 | 37.22 | 5.56 | 6.91 |
|  | 2015-2016 | 42.76 | 35.17 | 2.89 | 4.69 |
|  | 2019-2021 | 33.51 | 26.42 | 2.94 | 4.14 |
| **Puducherry** | 1998-1999 |  |  |  |  |
|  | 2005-2006 |  |  |  |  |
|  | 2015-2016 | 25.88 | 22.65 | 1.39 | 1.84 |
|  | 2019-2021 | 21.70 | 20.19 | 0.59 | 0.92 |
| **Andaman &  Nicobar Islands** | 1998-1999 |  |  |  |  |
|  | 2005-2006 |  |  |  |  |
|  | 2015-2016 | 55.64 | 20.01 | 18.77 | 16.85 |
|  | 2019-2021 | 20.26 | 16.96 | 2.50 | 0.80 |
| **Telangana** | 1998-1999 |  |  |  |  |
|  | 2005-2006 |  |  |  |  |
|  | 2015-2016 | 38.13 | 31.57 | 4.57 | 1.99 |
|  | 2019-2021 | 28.98 | 22.84 | 3.24 | 2.90 |

## **Table S3: Mortality Fractions (%) Attributable to smoking, smokeless and mixed tobacco use among women aged 35-49 years across 37 Indian states from 1998-1999 to 2019-2021.**

| **State** | **Year** | **Total Tobacco** | **Smoking** | **Smokeless** | **Mixed-Use** |
| --- | --- | --- | --- | --- | --- |
| **India** | 1998-1999 | 7.37 | 1.17 | 5.51 | 0.69 |
|  | 2005-2006 | 6.95 | 1.38 | 5.14 | 0.42 |
|  | 2015-2016 | 4.25 | 0.71 | 3.38 | 0.17 |
|  | 2019-2021 | 2.51 | 0.30 | 2.14 | 0.06 |
| **Jammu Kashmir** | 1998-1999 | 6.12 | 5.63 | 0.14 | 0.36 |
|  | 2005-2006 | 1.51 | 0.67 | 0.84 | 0.00 |
|  | 2015-2016 | 2.53 | 1.94 | 0.51 | 0.08 |
|  | 2019-2021 | 1.13 | 0.95 | 0.13 | 0.05 |
| **Himachal Pradesh** | 1998-1999 | 1.21 | 1.02 | 0.18 | 0.00 |
|  | 2005-2006 | 1.04 | 1.00 | 0.04 | 0.00 |
|  | 2015-2016 | 0.42 | 0.38 | 0.03 | 0.00 |
|  | 2019-2021 | 0.16 | 0.13 | 0.04 | 0.00 |
| **Punjab** | 1998-1999 | 0.29 | 0.15 | 0.08 | 0.06 |
|  | 2005-2006 | 0.69 | 0.44 | 0.19 | 0.06 |
|  | 2015-2016 | 0.18 | 0.09 | 0.04 | 0.05 |
|  | 2019-2021 | 0.09 | 0.05 | 0.04 | 0.00 |
| **Chandigarh** | 1998-1999 |  |  |  |  |
|  | 2005-2006 |  |  |  |  |
|  | 2015-2016 | 0.30 | 0.18 | 0.12 | 0.00 |
|  | 2019-2021 | 0.00 | 0.00 | 0.00 | 0.00 |
| **Uttarakhand** | 1998-1999 |  |  |  |  |
|  | 2005-2006 | 5.38 | 3.33 | 1.81 | 0.24 |
|  | 2015-2016 | 3.43 | 1.80 | 1.29 | 0.34 |
|  | 2019-2021 | 1.18 | 0.66 | 0.46 | 0.06 |
| **Haryana** | 1998-1999 | 2.67 | 1.87 | 0.42 | 0.38 |
|  | 2005-2006 | 3.56 | 2.98 | 0.43 | 0.14 |
|  | 2015-2016 | 1.57 | 1.33 | 0.22 | 0.03 |
|  | 2019-2021 | 0.62 | 0.51 | 0.11 | 0.01 |
| **Delhi** | 1998-1999 | 2.50 | 1.08 | 1.29 | 0.13 |
|  | 2005-2006 | 2.50 | 1.29 | 1.06 | 0.15 |
|  | 2015-2016 | 1.52 | 0.59 | 0.83 | 0.11 |
|  | 2019-2021 | 1.03 | 0.45 | 0.50 | 0.07 |
| **Rajasthan** | 1998-1999 | 4.85 | 2.40 | 2.18 | 0.27 |
|  | 2005-2006 | 5.49 | 2.94 | 2.30 | 0.24 |
|  | 2015-2016 | 3.96 | 1.70 | 2.15 | 0.12 |
|  | 2019-2021 | 2.64 | 0.94 | 1.61 | 0.09 |
| **Uttar Pradesh** | 1998-1999 | 7.46 | 1.38 | 5.07 | 1.01 |
|  | 2005-2006 | 8.93 | 2.36 | 5.81 | 0.75 |
|  | 2015-2016 | 6.11 | 1.47 | 4.20 | 0.45 |
|  | 2019-2021 | 2.60 | 0.48 | 2.01 | 0.11 |
| **Ladakh** | 1998-1999 |  |  |  |  |
|  | 2005-2006 |  |  |  |  |
|  | 2015-2016 |  |  |  |  |
|  | 2019-2021 | 2.02 | 1.53 | 0.34 | 0.15 |
| **Bihar** | 1998-1999 | 8.11 | 3.13 | 2.79 | 2.19 |
|  | 2005-2006 | 9.18 | 5.56 | 2.34 | 1.28 |
|  | 2015-2016 | 2.95 | 1.69 | 1.02 | 0.24 |
|  | 2019-2021 | 1.10 | 0.56 | 0.48 | 0.06 |
| **West Bengal** | 1998-1999 | 9.28 | 1.14 | 7.12 | 1.03 |
|  | 2005-2006 | 9.31 | 1.30 | 7.43 | 0.58 |
|  | 2015-2016 | 5.62 | 0.37 | 5.05 | 0.20 |
|  | 2019-2021 | 3.23 | 0.11 | 3.04 | 0.07 |
| **Jharkhand** | 1998-1999 |  |  |  |  |
|  | 2005-2006 | 7.62 | 0.81 | 6.23 | 0.57 |
|  | 2015-2016 | 3.96 | 0.23 | 3.66 | 0.07 |
|  | 2019-2021 | 1.88 | 0.31 | 1.48 | 0.09 |
| **Odisha** | 1998-1999 | 15.65 | 0.24 | 14.99 | 0.43 |
|  | 2005-2006 | 16.63 | 0.64 | 15.14 | 0.84 |
|  | 2015-2016 | 10.47 | 0.29 | 9.92 | 0.27 |
|  | 2019-2021 | 7.27 | 0.10 | 7.11 | 0.06 |
| **Sikkim** | 1998-1999 | 16.09 | 3.95 | 8.41 | 3.73 |
|  | 2005-2006 | 13.44 | 3.94 | 8.11 | 1.39 |
|  | 2015-2016 | 4.61 | 1.62 | 2.62 | 0.38 |
|  | 2019-2021 | 5.12 | 1.74 | 3.07 | 0.31 |
| **Arunachal Pradesh** | 1998-1999 | 21.20 | 2.71 | 13.04 | 5.44 |
|  | 2005-2006 | 17.52 | 2.27 | 12.16 | 3.09 |
|  | 2015-2016 | 10.98 | 1.90 | 7.73 | 1.36 |
|  | 2019-2021 | 6.74 | 1.22 | 4.90 | 0.62 |
| **Nagaland** | 1998-1999 | 6.85 | 0.27 | 5.62 | 0.96 |
|  | 2005-2006 | 8.43 | 0.33 | 7.90 | 0.20 |
|  | 2015-2016 | 9.27 | 0.10 | 9.06 | 0.11 |
|  | 2019-2021 | 5.17 | 0.04 | 5.13 | 0.00 |
| **Manipur** | 1998-1999 | 17.69 | 4.80 | 7.67 | 5.22 |
|  | 2005-2006 | 24.78 | 4.49 | 14.10 | 6.19 |
|  | 2015-2016 | 23.29 | 1.28 | 18.37 | 3.64 |
|  | 2019-2021 | 19.67 | 0.50 | 17.76 | 1.41 |
| **Mizoram** | 1998-1999 | 51.45 | 4.64 | 19.36 | 27.45 |
|  | 2005-2006 | 47.40 | 10.56 | 18.08 | 18.76 |
|  | 2015-2016 | 44.30 | 8.69 | 18.77 | 16.84 |
|  | 2019-2021 | 27.46 | 4.07 | 16.81 | 6.58 |
| **Tripura** | 1998-1999 | 7.40 | 4.50 | 2.03 | 0.87 |
|  | 2005-2006 | 38.55 | 6.60 | 18.62 | 13.34 |
|  | 2015-2016 | 25.47 | 3.00 | 18.01 | 4.47 |
|  | 2019-2021 | 21.30 | 1.58 | 17.16 | 2.56 |
| **Meghalaya** | 1998-1999 | 17.78 | 3.44 | 12.58 | 1.76 |
|  | 2005-2006 | 15.97 | 1.94 | 13.10 | 0.93 |
|  | 2015-2016 | 16.03 | 2.03 | 12.57 | 1.44 |
|  | 2019-2021 | 11.70 | 0.49 | 10.64 | 0.57 |
| **Assam** | 1998-1999 | 14.27 | 0.58 | 11.19 | 2.50 |
|  | 2005-2006 | 14.41 | 0.63 | 12.74 | 1.04 |
|  | 2015-2016 | 10.20 | 0.16 | 9.83 | 0.22 |
|  | 2019-2021 | 7.95 | 0.44 | 7.27 | 0.24 |
| **Chhattisgarh** | 1998-1999 |  |  |  |  |
|  | 2005-2006 | 8.68 | 0.29 | 8.07 | 0.33 |
|  | 2015-2016 | 4.84 | 0.13 | 4.59 | 0.12 |
|  | 2019-2021 | 4.01 | 0.12 | 3.82 | 0.07 |
| **Madhya Pradesh** | 1998-1999 | 7.45 | 0.45 | 6.68 | 0.32 |
|  | 2005-2006 | 8.31 | 0.51 | 7.45 | 0.35 |
|  | 2015-2016 | 5.62 | 0.48 | 5.03 | 0.10 |
|  | 2019-2021 | 3.41 | 0.14 | 3.23 | 0.04 |
| **Gujarat** | 1998-1999 | 4.17 | 0.50 | 3.53 | 0.14 |
|  | 2005-2006 | 5.40 | 0.59 | 4.58 | 0.23 |
|  | 2015-2016 | 3.90 | 0.21 | 3.60 | 0.09 |
|  | 2019-2021 | 2.78 | 0.23 | 2.51 | 0.03 |
| **Dadra &  Nagar Haveli** | 1998-1999 |  |  |  |  |
|  | 2005-2006 |  |  |  |  |
|  | 2015-2016 | 1.41 | 0.10 | 1.31 | 0.00 |
|  | 2019-2021 | 0.67 | 0.00 | 0.67 | 0.00 |
| **Daman & Diu** | 1998-1999 |  |  |  |  |
|  | 2005-2006 |  |  |  |  |
|  | 2015-2016 | 0.53 | 0.00 | 0.53 | 0.00 |
|  | 2019-2021 |  |  |  |  |
| **Maharashtra** | 1998-1999 | 9.07 | 0.03 | 8.97 | 0.06 |
|  | 2005-2006 | 5.97 | 0.06 | 5.82 | 0.09 |
|  | 2015-2016 | 3.38 | 0.20 | 3.15 | 0.03 |
|  | 2019-2021 | 2.02 | 0.07 | 1.93 | 0.02 |
| **Andhra Pradesh** | 1998-1999 | 7.78 | 2.27 | 4.64 | 0.87 |
|  | 2005-2006 | 4.06 | 0.82 | 3.24 | 0.00 |
|  | 2015-2016 | 1.78 | 0.89 | 0.86 | 0.03 |
|  | 2019-2021 | 0.68 | 0.08 | 0.57 | 0.04 |
| **Karnataka** | 1998-1999 | 6.77 | 0.08 | 6.69 | 0.00 |
|  | 2005-2006 | 3.58 | 0.16 | 3.31 | 0.11 |
|  | 2015-2016 | 3.10 | 0.49 | 2.45 | 0.16 |
|  | 2019-2021 | 1.96 | 0.28 | 1.65 | 0.04 |
| **Goa** | 1998-1999 | 4.07 | 0.46 | 3.50 | 0.11 |
|  | 2005-2006 | 2.93 | 0.16 | 2.72 | 0.05 |
|  | 2015-2016 | 1.14 | 0.07 | 1.07 | 0.00 |
|  | 2019-2021 | 0.56 | 0.13 | 0.43 | 0.00 |
| **Lakshadweep** | 1998-1999 |  |  |  |  |
|  | 2005-2006 |  |  |  |  |
|  | 2015-2016 | 10.34 | 2.44 | 7.90 | 0.00 |
|  | 2019-2021 | 3.22 | 0.91 | 2.31 | 0.00 |
| **Kerala** | 1998-1999 | 2.88 | 0.10 | 2.73 | 0.06 |
|  | 2005-2006 | 1.12 | 0.09 | 1.03 | 0.00 |
|  | 2015-2016 | 0.44 | 0.00 | 0.44 | 0.00 |
|  | 2019-2021 | 0.21 | 0.01 | 0.20 | 0.00 |
| **Tamil Nadu** | 1998-1999 | 5.29 | 0.23 | 5.02 | 0.04 |
|  | 2005-2006 | 2.04 | 0.05 | 1.99 | 0.00 |
|  | 2015-2016 | 1.65 | 0.09 | 1.55 | 0.01 |
|  | 2019-2021 | 0.82 | 0.10 | 0.70 | 0.02 |
| **Puducherry** | 1998-1999 |  |  |  |  |
|  | 2005-2006 |  |  |  |  |
|  | 2015-2016 | 0.94 | 0.00 | 0.94 | 0.00 |
|  | 2019-2021 | 0.16 | 0.00 | 0.16 | 0.00 |
| **Andaman &  Nicobar Islands** | 1998-1999 | 0.00 |  |  |  |
|  | 2005-2006 | 0.00 |  |  |  |
|  | 2015-2016 | 12.63 | 0.13 | 12.25 | 0.26 |
|  | 2019-2021 | 8.45 | 0.07 | 8.37 | 0.01 |
| **Telangana** | 1998-1999 |  |  |  |  |
|  | 2005-2006 |  |  |  |  |
|  | 2015-2016 | 2.24 | 0.47 | 1.69 | 0.08 |
|  | 2019-2021 | 1.42 | 0.22 | 1.15 | 0.04 |

## **Table S4: Deaths (in million) attributable to tobacco use type among men (35-54 years) in India across states during 2019-2021.**

| **State^*^** | **Smoking** | **Smokeless** | **Mixed-Use** | **Total Tobacco** |
| --- | --- | --- | --- | --- |
| **India** | **0.288066** | **0.098765** | **0.082305** | **0.469136** |
| Jammu and Kashmir | 0.000095 | 0.000004 | 0.000010 | 0.000109 |
| Himachal Pradesh | 0.001808 | 0.000140 | 0.000265 | 0.002213 |
| Punjab | 0.005615 | 0.000776 | 0.000848 | 0.007239 |
| Chandigarh | 0.000866 | 0.000156 | 0.000157 | 0.001179 |
| Uttarakhand | 0.003068 | 0.000881 | 0.001253 | 0.005202 |
| Haryana | 0.011980 | 0.000593 | 0.000773 | 0.013347 |
| Delhi | 0.007336 | 0.001433 | 0.001804 | 0.010573 |
| Rajasthan | 0.022758 | 0.006001 | 0.008005 | 0.036764 |
| Uttar Pradesh | 0.030351 | 0.014068 | 0.016015 | 0.060433 |
| Ladakh | 0.000012 | 0.000001 | 0.000002 | 0.000016 |
| Bihar | 0.012007 | 0.010701 | 0.009058 | 0.031766 |
| West Bengal | 0.030236 | 0.005787 | 0.009345 | 0.045368 |
| Odisha | 0.008792 | 0.005583 | 0.004110 | 0.018485 |
| Arunachal Pradesh | 0.000313 | 0.000106 | 0.000154 | 0.000573 |
| Nagaland | 0.000179 | 0.000072 | 0.000085 | 0.000336 |
| Mizoram | 0.000677 | 0.000120 | 0.000340 | 0.001137 |
| Manipur | 0.000105 | 0.000042 | 0.000061 | 0.000208 |
| Tripura | 0.000965 | 0.000137 | 0.000122 | 0.001225 |
| Meghalaya | 0.001373 | 0.000132 | 0.000409 | 0.001914 |
| Assam | 0.005977 | 0.002373 | 0.002823 | 0.011173 |
| Chhattisgarh | 0.007589 | 0.003207 | 0.003321 | 0.014118 |
| Madhya Pradesh | 0.020406 | 0.009227 | 0.008614 | 0.038247 |
| Gujarat | 0.013624 | 0.009212 | 0.004638 | 0.027474 |
| Daman & Diu and Dadra and Nagar Haveli | 0.000202 | 0.000041 | 0.000050 | 0.000293 |
| Andhra Pradesh | 0.011418 | 0.001034 | 0.000914 | 0.013366 |
| Karnataka | 0.018949 | 0.003716 | 0.002756 | 0.025421 |
| Goa | 0.000442 | 0.000102 | 0.000070 | 0.000614 |
| Lakshadweep | 0.000009 | 0.000002 | 0.000002 | 0.000014 |
| Kerala | 0.004853 | 0.000323 | 0.000596 | 0.005772 |
| Tamil Nadu | 0.022195 | 0.002473 | 0.003481 | 0.028150 |
| Puducherry | 0.000408 | 0.000012 | 0.000019 | 0.000439 |
| Andaman & Nicobar Island | 0.000078 | 0.000011 | 0.000004 | 0.000093 |
| Telangana | 0.003581 | 0.000508 | 0.000454 | 0.004543 |

^*^Note: State of Maharashtra, Jharkhand and Sikkim have not been included due to partial/incomplete death data.

## **Table S5: Deaths (in million) attributable to tobacco use type among women (35-49 years) in India across states during 2019-2021.**

| **State^*^** | **Smoking** | **Smokeless** | **Mixed-Use** | **Total Tobacco** |
| --- | --- | --- | --- | --- |
| **India** | **0.001107** | **0.007751** | **0.000369** | **0.009228** |
| Jammu Kashmir | 0.000002 | 0.000000 | 0.000000 | 0.000002 |
| Himachal Pradesh | 0.000002 | 0.000001 | 0.000000 | 0.000003 |
| Punjab | 0.000008 | 0.000007 | 0.000000 | 0.000015 |
| Chandigarh | 0.000000 | 0.000000 | 0.000000 | 0.000000 |
| Uttarakhand | 0.000027 | 0.000019 | 0.000003 | 0.000049 |
| Haryana | 0.000051 | 0.000011 | 0.000001 | 0.000063 |
| Delhi | 0.000045 | 0.000050 | 0.000007 | 0.000101 |
| Rajasthan | 0.000263 | 0.000454 | 0.000025 | 0.000742 |
| Uttar Pradesh | 0.000281 | 0.001170 | 0.000062 | 0.001514 |
| Ladakh | 0.000000 | 0.000000 | 0.000000 | 0.000001 |
| Bihar | 0.000190 | 0.000163 | 0.000019 | 0.000373 |
| West Bengal | 0.000048 | 0.001272 | 0.000029 | 0.001349 |
| Odisha | 0.000025 | 0.001727 | 0.000014 | 0.001765 |
| Arunachal Pradesh | 0.000004 | 0.000016 | 0.000002 | 0.000022 |
| Nagaland | 0.000000 | 0.000011 | 0.000000 | 0.000011 |
| Mizoram | 0.000022 | 0.000089 | 0.000035 | 0.000145 |
| Manipur | 0.000000 | 0.000018 | 0.000001 | 0.000019 |
| Tripura | 0.000005 | 0.000026 | 0.000001 | 0.000033 |
| Meghalaya | 0.000007 | 0.000149 | 0.000008 | 0.000163 |
| Assam | 0.000053 | 0.000874 | 0.000029 | 0.000955 |
| Chhattisgarh | 0.000017 | 0.000532 | 0.000010 | 0.000559 |
| Madhya Pradesh | 0.000049 | 0.001128 | 0.000013 | 0.001190 |
| Gujarat | 0.000065 | 0.000714 | 0.000009 | 0.000788 |
| Daman & Diu and Dadra and Nagar Haveli | 0.000002 | 0.000004 | 0.000000 | 0.000006 |
| Andhra Pradesh | 0.000015 | 0.000103 | 0.000007 | 0.000125 |
| Karnataka | 0.000092 | 0.000546 | 0.000013 | 0.000651 |
| Goa | 0.000001 | 0.000003 | 0.000000 | 0.000003 |
| Lakshadweep | 0.000000 | 0.000000 | 0.000000 | 0.000000 |
| Kerala | 0.000001 | 0.000017 | 0.000000 | 0.000018 |
| Tamil Nadu | 0.000037 | 0.000269 | 0.000007 | 0.000314 |
| Puducherry | 0.000000 | 0.000001 | 0.000000 | 0.000001 |
| Andaman & Nicobar Island | 0.000000 | 0.000017 | 0.000000 | 0.000017 |
| Telangana | 0.000028 | 0.000145 | 0.000006 | 0.000179 |

^*^Note: State of Maharashtra, Jharkhand and Sikkim have not been included due to partial/incomplete death data.
